# Factors affecting the stability and repeatability of gamma camera calibration for quantitative imaging applications based on a retrospective review of clinical data

**DOI:** 10.1186/s13550-014-0067-x

**Published:** 2014-12-16

**Authors:** Nadège Anizan, Hao Wang, Xian C Zhou, Robert F Hobbs, Richard L Wahl, Eric C Frey

**Affiliations:** The Russell H. Morgan Department of Radiology and Radiological Science, Johns Hopkins University, 601 North Caroline Street/JHOC 4263, Baltimore, Maryland 21287 USA; Department of Oncology, Johns Hopkins University, Baltimore, MD USA; Department of Radiation Oncology, Johns Hopkins University, Baltimore, MD USA

**Keywords:** Gamma camera, Calibration, Absolute activity quantification, Repeatability

## Abstract

**Background:**

Absolute quantitative single-photon emission computed tomography (SPECT) has several important applications including monitoring tumor response after treatment and dose estimation for targeted radionuclide therapy treatment planning. Obtaining quantitative SPECT images in absolute activity units requires the use of a calibration factor, and the repeatability of this directly affects the repeatability of image quantification. This study focused on evaluating the factors affecting the repeatability of a calibration factor measured using a planar image of an in-air calibration source.

**Methods:**

The calibration factors calculated as part of ^131^I-tositumomab patient dosimetry scans used in treatment planning performed over a 4-year period were retrospectively analyzed. Raw data included total counts in whole-body images of a radioactive calibration source, the activity of the source measured in a radionuclide activity meter (often referred to as a dose calibrator), and the background count rate obtained at three time points for each patient. The count rate from extrinsic flood source acquisitions and radionuclide activity meter constancy obtained on the same day as each image were also used. The data were analyzed statistically using a mixed-effects model to determine the factors affecting variations in the measured calibration factors.

**Results:**

The global variability in the calibration factor was equal to 2.3% and was decreased by 20% to 1.8%, when the decay-corrected measurements of calibration source activity were averaged over the three time points for each patient. Camera sensitivity variation measured using a ^57^Co sheet source was small and had a weak relationship to calibration factor variations. When the averaged source activity was used, the main source of variance was related to preparation and measurement of the source (77%). Radionuclide activity meter constancy had a smaller but statistically significant impact on the calibration factor.

**Conclusions:**

This study indicates that calibration factors based on planar measurements have good reproducibility. The findings of this study indicate (1) the importance of accurate and precise preparation and measurement of the calibration source activity, (2) the need to carefully control background activity during calibration factor assessment and patient data acquisition, and (3) that the calibration factor and camera sensitivity were stable over time, indicating that careful but less frequent calibration is needed.

## Background

Absolute activity quantification is an important task in a number of nuclear medicine applications including radionuclide therapy dosimetry for absorbed dose-based treatment planning [1,2] and for serial quantitative imaging to assess tumor progression or response to therapy [3]. Accurate quantification of single-photon emission computed tomography (SPECT)/CT images requires compensation for all physical processes, such as attenuation, scatter, and partial volume effects [4], and a precise conversion of the voxel values in the image to activity using a calibration factor. Zeintl et al. [5] showed that the uncertainties in the calibration factor are a dominant factor in the variability of absolute quantification in SPECT imaging. Several methods have been used in the literature to determine this calibration factor: simulation of an in-air point source [6] or imaging of a cylindrical phantom [7], an elliptical tank without [5,6] or with a hot sphere placed inside [8], a set of small sources of different sizes imaged in air followed by extrapolation to a point source [9], and a syringe [10] using SPECT/CT or planar acquisition. Because of the wide range in the methods proposed, both IAEA Human Health Reports No. 9 [11] and MIRD Pamphlet 23 made some recommendations about calibration procedures, the latter in the context of dosimetry calculations for internal radionuclide therapy using SPECT/CT imaging. Depending on the accuracy of the corrections applied as part of the SPECT/CT imaging reconstruction process, the user has the choice between a planar image of a small source or a SPECT/CT acquisition of a phantom uniformly filled with activity with optional inclusion of a hot sphere placed inside. Because of the simplicity of the in-air source measurement, Frey et al. [12] recommended the first method when attenuation, scatter, and collimator-detector response are accurately compensated for in the SPECT reconstruction process. The simplicity of this approach has some potential advantages in terms of repeatability and is thus well suited to routine monitoring for changes in the calibration factor.

In the context of absorbed dose calculation for targeted radionuclide therapy or for the evaluation of the tumor response to therapy, it is important that the measurement be repeatable and the factor stable over time in order to provide accurate absolute activity quantification. However, the repeatability of the calibration factor measurement and its stability over time have not been carefully investigated, and little information can be found in the literature about factors impacting its variability. Thus, we studied the variability of the calibration factor measurement using data from a series of patient ^131^I-tositumomab + unlabeled tositumomab (Bexxar®, GlaxoSmithKline, Brentford, England) radionuclide therapy procedures and quality control measurements obtained over a period of 4 years. The calibration factor was measured by a dual-camera whole-body anterior-posterior scan of an in-air calibration source and was performed for each patient acquisition. This served as the raw data on calibration factor stability and repeatability. This work focused on the assessment of the repeatability of this in-air calibration factor measurement over a long time period and on the factors affecting its repeatability by analyzing sources of variability. One factor evaluated was the quantitative stability of the SPECT camera over time, which has a direct effect on the stability of the calibration factor. As a proxy for this, we used the count rate measured in daily extrinsic ^57^Co flood acquisitions performed as part of a routine quality control program. Since this was measured using a different radionuclide and collimator, it assesses only the radionuclide and collimator independent component of camera sensitivity variations. Since the activities in the calibration sources used for calibration were measured using a radionuclide activity meter, radionuclide activity meter variability may also impact the calibration factor calculation. To assess this, we used results of daily radionuclide activity meter constancy measurements. Finally, the variable radiation environment in the SPECT camera room may also be a factor affecting the variability of the calibration factor measurement. To quantify this effect, we used background measurements routinely acquired as part of the radionuclide therapy protocol. Using these data, we performed a number of statistical analyses to evaluate the importance of the various potential sources of variation and the overall stability of the calibration factor.

## Methods

### Overview

The data used came from imaging studies performed for dosimetry calculations for 46 patients from 2007 to 2011 treated with the Bexxar® therapeutic regimen for non-Hodgkin’s [13] and Hodgkin’s lymphoma. In order to quantify the drug distribution obtained by planar whole-body scans, a calibration factor (in units of cps·MBq^−1^) was measured using a vial containing ^131^I-tositumomab imaged in air before each patient image acquisition. The calibration source was prepared for each patient and its activity was measured using the radionuclide activity meter described below before each calibration scan. In addition to the data from the calibration scans, radionuclide activity meter variability was assessed using data from daily constancy tests, and camera variability was evaluated using count rates from daily extrinsic flood quality control acquisitions. All the data were analyzed using a statistical mixed-effects model [14], described in more detail below, in order to determine the global variability of the calibration factor and the magnitude of components of its variance due to various factors.

### Calibration factor

The calibration factor was defined as the ratio between the total background-corrected count rate in the whole image and the true total activity of ^131^I-tositumomab (7.5 to 10.8 MBq) in a 10-ml vial that served as a calibration source. For each patient, the same source was used on all 3 days of the patient acquisition. The source was placed at the center of the camera field of view, and a whole-body acquisition of the vial and a background acquisition were obtained before each patient image acquisition on the day (D0) of the radiopharmaceutical injection and 3 (D3) and 6 days (D6) post-injection using the same acquisition parameters as for the whole-body patient image. Data were acquired using a dual camera Millennium VG SPECT camera system with Hawkeye CT (GE Healthcare), resulting in a total of 137 acquisitions (one patient had data at only two time points). All acquisitions were performed with a high-energy general-purpose (HEGP) collimator, a 20% energy window centered at 364 keV, a 15 cm·min^−1^ bed speed, and a scan length of either 198 or 196 cm. The calibration factor, CF, was calculated using:

1 $$ \mathrm{C}\mathrm{F}\kern0.5em =\kern0.5em \frac{\mathrm{Calibration}\kern0.5em \mathrm{source}\kern0.5em \mathrm{count}\kern0.5em \mathrm{rate}\kern0.5em -\kern0.5em \mathrm{Background}\kern0.5em \mathrm{count}\kern0.5em \mathrm{rate}}{\mathrm{Calibration}\kern0.5em \mathrm{source}\kern0.5em \mathrm{activity}} $$

The total calibration source count rate and the total background count rate were calculated as the average counts over the two detectors in the whole-body image divided by the acquisition time. The calibration source activity was measured using a radionuclide activity meter and corrected for radioactive decay. Because the data suggested that variability in measuring the calibration source’s activity in the radionuclide activity meter was a significant source of variability in the calibration factor, we calculated the calibration factor using values of the calibration source activity term in Equation 1 calculated in two different ways. In both cases, the calibration source activity was measured on each day of the calibration source acquisition. In the first method (CF_rA_), we used the calibration source activity measured on the day of the acquisition as value of the calibration source activity term; in the second method (CF_dA_), we used the average of the decay-corrected activity measured over the 3 days.

### Radionuclide activity meter

The ^131^I-tositumomab activity in the vial was measured using a CRC®-15R (Capintec, Inc., Ramsey, NJ, USA) radionuclide activity meter on D0, D3, and D6. The radionuclide activity meter was originally calibrated by the manufacturer. A constancy test for this calibrator was performed every morning as part of routine quality control using a ^57^Co source where the read activity value was compared to the predicted value calculated by accounting for radioactive decay. From 2007 to 2011, three ^57^Co sources, each from a different vendor, were used for the test. The initial activities of these sources (185, 185, and 370 MBq) were provided by the vendors and were traceable to NIST standards. Note that the results of this study would not be affected by the accuracy of the activity meter calibration as only the variability of the calibration factor was studied. In addition, only the constancy, the difference between the indicated and true activity of the calibration source, was used in the statistical analysis. Records of the accuracy of the activity of the sources used for the constancy test are not available. However, current sources were purchased from Eckert & Ziegler (Berlin, Germany) and have an accuracy of ±5%. A plot of the constancy values over time showed no discontinuities due to source changes. A reproducibility test (ten successive measurements [15]) performed with the same ^57^Co source showed a variability of 0.1% of the calibration source activity measurement for this radionuclide activity meter.

### Extrinsic flood acquisition

During this period, a ^57^Co sheet source was imaged daily with the system’s low-energy high-resolution (LEHR) collimators. A static acquisition of the sheet source placed between the two cameras and as close as possible to the collimators was performed and stopped when 10^6^ counts per camera were collected. We used this data as a measure of variability of the global sensitivity since this source has a long life and is large, so that it covers the useful field of view of the camera. The count rate (10^6^/acquisition time) averaged over the two cameras was divided by the decay-corrected known activity of the source to calculate the sensitivity factor (*S*_EF_). Throughout the 4 years of data acquisition, six different flood sources were used. Data on the accuracy activities of the flood sources is not available. However, the commercial sheet sources used at present have a coefficient of variation of ±1% and an integral uniformity of 3.6% according to the manufacturer (FeatherLite, Eckert & Ziegler). Note that since the count rate was estimated over a large region of interest, the uniformity of the sheet source would have a small effect on sensitivity variations due to variations in source position. In addition, since the sheet source count rate was only used as an indicator of the stability of camera sensitivity, the only impact of the accuracy of the specified flood source activity would be discontinuities in the count rate from the ^57^Co source when a new source is used.

The value of *S*_EF_ was corrected for count loss due to dead time using a paralyzable model (Equation 2). The dead time, *τ*, was estimated based on the fitting observed count rate at each acquisition time, *t*, as a function of the activity of the ^57^Co sheet source, *A*(*t*), at time *t*, with the equation describing paralyzable count rate behavior:

2 $$ \mathrm{Observed}\kern0.5em \mathrm{count}\kern0.5em \mathrm{rate}(t)\kern0.5em =\kern0.5em C\kern0.5em \times \kern0.5em A\kern0.5em \times \kern0.5em  \exp \left(-\tau \kern1em \times \kern0.5em C\kern0.5em \times \kern0.5em A(t)\right) $$

In Equation 2, *C* is a constant estimated in the fitting that relates the activity to the count rate incident on the camera, and the estimated dead time was equal to 5.5 μs.

### Statistical analysis

The purpose of the statistical analysis was to investigate the magnitudes of the components of variance of the calibration factor and to assess their impact on calibration factor variability. To do this, we used a statistical methodology that models the relationship between explanatory variables (such as *S*_EF_, radionuclide activity meter constancy, and background count rate) and dependent variables (such as CF_rA_ and CF_dA_). Similarly, we studied the influence of *S*_EF_ and background count rate on the measured calibration source count rate, as well as the impact of *S*_EF_ on the background count rate. We used a mixed-effects model [14] where some explanatory variables were modeled as fixed effects and some as random effects. A fixed effect is one where there is a functional relationship between the explanatory variable and quantity of interest; in this work, we assumed the relationship to be linear. For example, we assumed that there was a linear relationship between the camera sensitivity factor and the calibration factor for reasons described below. A random effect is one where the contribution from that variable for a particular measurement is a sample of a random variable; in this work, all the random variables were assumed to have zero-mean normal distributions with unknown variances.

We used a different statistical model for each of the three quantities of interest, as shown in Table 1 and described in detail below. The first model, and the main focus of this paper, was used to analyze the sources of variation in the calibration factor itself. The calibration factor depends on three terms: the calibration source count rate, the background count rate, and the activity of the calibration source. It is also desirable to understand sources of variation in these measurements. We thus used two additional statistical models to investigate this. Models 2 and 3 were used to study the factors affecting the background and calibration source count rates, respectively. A statistical model was not used to study the calibration source activity measurement. However, as described above, we used two methods to calculate the calibration source activity used in the calibration factor calculation.

**Table 1:** **Mixed-effects models used for the different variables**

**Model**	**Outcome variable**	**Model definition**
1	Calibration factors CF_rA_ and CF_dA_	$y\_\mathrm{C}{\mathrm{F}}_{ik}\kern0.5em =\kern0.5em {\mu}_{\mathrm{CF}}\kern0.5em +\kern0.5em \alpha \_\mathrm{C}{\mathrm{F}}_i\kern0.5em +\kern0.5em {\beta}_{S_{\mathrm{E}\mathrm{F}}}\kern0.5em \times \kern0.5em {S}_{\mathrm{E}{\mathrm{F}}_{ik}}\kern0.5em +\kern0.5em {\beta}_{\mathrm{Constancy}}\kern0.5em \times \kern0.5em \mathrm{Constanc}{\mathrm{y}}_{ik}\kern0.5em +\kern0.5em {\gamma}_{\mathrm{Bk}{\mathrm{R}}_i}\kern0.5em \times \kern0.5em \mathrm{B}\mathrm{k}{\mathrm{R}}_{ik}\kern0.5em +\kern0.5em {\varepsilon}_{ik}$ for calibration source *i* on day *k*, with $\alpha \_\mathrm{C}{\mathrm{F}}_i\kern0.5em \sim \kern0.5em N\left(0,{\sigma}_{\mathrm{CF}}^2\right)$, ${\gamma}_{\mathrm{Bk}{\mathrm{R}}_i}\kern0.5em \sim \kern0.5em N\left(0,{\sigma}_{\gamma_{\mathrm{Bk}\mathrm{R}}}^2\right)$, and ${\varepsilon}_{ik}\kern0.5em \sim \kern0.5em N\left(0,{\sigma}_{\varepsilon}^2\right)$
2	Background count rate	$y\_\mathrm{B}\mathrm{k}{\mathrm{R}}_{ik}\kern0.5em =\kern0.5em {\mu}_{\mathrm{BkR}}\kern0.5em +\kern0.5em \alpha \_\mathrm{B}\mathrm{k}{\mathrm{R}}_i\kern0.5em +\kern0.5em {\beta}_{S_{\mathrm{E}\mathrm{F}}}\kern0.5em \times \kern0.5em {S}_{\mathrm{E}{\mathrm{F}}_{ik}}\kern0.5em +\kern0.5em {\varepsilon}_{ik}$ for calibration source *i* on day *k*, with $\alpha \_\mathrm{B}\mathrm{k}{\mathrm{R}}_i\kern0.5em \sim \kern0.5em N\left(0,{\sigma}_{\mathrm{BkR}}^2\right)$, and ${\varepsilon}_{ik}\kern0.5em \sim \kern0.5em N\left(0,{\sigma}_{\varepsilon}^2\right)$
3	Calibration source count rate	$y\_\mathrm{S}{\mathrm{R}}_{ik}\kern0.5em =\kern0.5em {\mu}_{\mathrm{SR}}\kern0.5em +\kern0.5em \alpha \_\mathrm{S}{\mathrm{R}}_i\kern0.5em +\kern0.5em {\beta}_{S_{\mathrm{E}\mathrm{F}}}\kern0.5em \times \kern0.5em {S}_{\mathrm{E}{\mathrm{F}}_{ik}}\kern0.5em +\kern0.5em {\gamma}_{\mathrm{Bk}{\mathrm{R}}_i}\kern0.5em \times \kern0.5em \mathrm{B}\mathrm{k}{\mathrm{R}}_{ik}\kern0.5em +\kern0.5em {\varepsilon}_{ik}$ for calibration source *i* on day *k*, with $\alpha \_\mathrm{S}{\mathrm{R}}_i\kern0.5em \sim \kern0.5em N\left(0,{\sigma}_{\mathrm{SR}}^2\right)$, ${\gamma}_{\mathrm{Bk}{\mathrm{R}}_i}\kern0.5em \sim \kern0.5em N\left(0,{\sigma}_{\gamma_{\mathrm{Bk}\mathrm{R}}}^2\right)$, and ${\varepsilon}_{ik}\kern0.5em \sim \kern0.5em N\left(0,{\sigma}_{\varepsilon}^2\right)$

*y_*CF_*ik*_ denotes the standardized calibration factor for calibration source *i* and day *k.*

*μ*CF is the mean, and *α*_CF_*i*_ is the random intercept representing a contribution specific to calibration source *i*.

${\sigma}_{\mathrm{CF}}^2$ is the variance of the zero-mean normal distribution of the random intercept.

${\sigma}_{\gamma \mathrm{B}\mathrm{k}\mathrm{R}}^2$ is the variance of the zero-mean normal distribution representing the background count rate contribution.

${\sigma}_{\varepsilon}^2$ is the variance of the zero-mean normal distribution representing the residual error.

*S*_EF*ik*_ is the standardized camera sensitivity factor for source *i* and day *k.*

Constancy_*ik*_ is the standardized radionuclide activity meter constancy value for source *i* and day *k*.

BkR_*ik*_ is the standardized background count rate for source *i* and day *k*.

*y*_BkR_*ik*_ denotes the standardized background count rate for calibration source *i* and day *k*, *μ*BkR is the mean value, and *α*_BkR_*i*_ is the subject-specific effect.

*y*_SR_*ik*_ denotes the standardized calibration source count rate for calibration source *i* and day *k.*

*μ*_SR_ is the mean, and *α*_SR_*i*_ is the random intercept representing a contribution specific to calibration source *i*.

${\beta}_{S_{\mathrm{EF}}}$ is the slope of fixed effect representing the camera assessed using the standardized sensitivity factor.

*β*_Constancy_ is the slope of the fixed effect representing the radionuclide activity meter assessed using the standardized constancy.

${\gamma}_{BkR_{ik}}$ is the random effect of the standardized background count rate.

*ε*_*ik*_ is the residual error.

In each case, the model consisted of an unknown fixed intercept, a random intercept, and terms for each fixed or random effect. The random intercept, representing the variations across different calibration sources, was assumed to follow a normal distribution with a mean of zero and an unknown variance that was particular to each calibration source. We included terms that were proportional to the value of each fixed-effect variable, where the proportionality constant (slope) was unknown, and terms proportional to each random-effect variable, where the proportionality constant was a zero-mean normally distributed random variable with unknown variance. The model was fitted to the data, as described below, to give estimates of the unknown values, i.e., the mean, slopes for the fixed effects, and variances for the random effects.

Specifically, we evaluated the components of variance in the measurements of the calibration factors CF_rA_ and CF_dA_ using a mixed-effects model (Table 1, model 1). The calibration factors could, in principle, vary with camera sensitivity (*S*_EF_), radionuclide activity meter constancy, and background count rate. We included *S*_EF_ and constancy as fixed effects and background as a random effect, for reasons described below. We used a random intercept to represent variations between calibration sources since we considered them to be a random sample of calibration sources and were not interested in the specific effect of each source. The effect of dead time and pileup on the calibration factor calculation was considered negligible, as will be discussed in more detail below.

The calibration source count rate and the background count rate used to calculate the calibration factor were also evaluated separately using a mixed-effects model to estimate the influence of the previous listed effects on each step of the calibration factor calculation. The model for the background count rate (Table 1, model 2) treated the sensitivity factor as a fixed effect and the calibration source as a random effect. The model for the calibration source count rate (Table 1, model 3) considered the sensitivity factor as a fixed effect and the background count rate and calibration source as random effects.

We considered the sensitivity factor (*S*_EF_) to be a fixed effect because we assumed a systematic association between the calibration factor and the sensitivity factor over time. The extrinsic flood was always acquired using a large number of counts (10^6^), leading to a very low Poisson noise level in the sensitivity factor $\left(\sqrt{\mu }/\mu \kern0.5em =\kern0.5em 0.1\%\right)$; the random effect of this noise was ignored. The radionuclide activity meter constancy was also assumed to be a fixed effect as we assumed a systematic association between the calibration factor and the radionuclide activity meter constancy. A repeatability of 0.1% of the measurement of a standard source activity was found for this radionuclide activity meter, justifying ignoring random effects in this measurement. Because of the small number of counts obtained during the background acquisition and the unpredictable radiation environment in the SPECT camera room, the background count rate was considered a random effect.

We used restricted maximum likelihood (REML) [14] estimation to estimate the parameters of the fixed effects and the random effects in the models. To simplify the interpretation of the results of the analysis with variables on very different scales, all variables (CF_rA_, CF_dA_, *S*_EF_, radionuclide activity meter constancy, calibration source count rate, and background count rate) were standardized by first subtracting their mean and then dividing by their standard deviation. In this way, all variables were in the same scale with a mean of 0 and a variance of 1.

## Results

**Figure 1:**
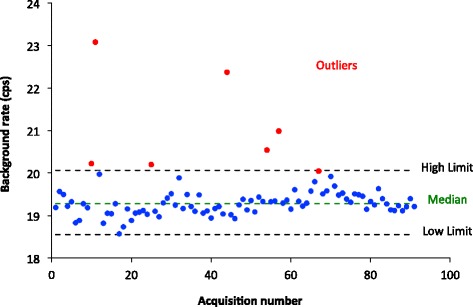
**Background count rate distribution.** The red data points indicate outliers excluded as described in the text. The blue data points were used in the statistical analysis.

All time points for which a value of the calibration factor, radionuclide activity meter constancy, or intrinsic flood was not all available were excluded from the analysis, leading to 91 acquisitions for 41 calibration sources. In addition, seven points were removed because of an abnormally high background count rate value, possibly due to the presence of radiation in or around the room during the background acquisition, which might not have been present during the calibration acquisition. Those seven points were excluded as outliers (see Figure 1) based on the criterion that the remaining data represent 99.3% of a normally distributed variable:

1. The high limit was defined as Quartile_0.75_ + 1.5 × (Quartile_0.75_ − Quartile_0.25_)
2. The low limit was defined as Quartile_0.25_ − 1.5 × (Quartile_0.75_ − Quartile_0.25_)

After excluding the above points, there were data from a total of 41 calibration sources used in the study, with 12 having three time points, 19 having two time points, and 10 with only one time point, representing a total of 84 calibration source acquisitions.

### Calibration factor using the read activity: CF_rA_

**Figure 2:**
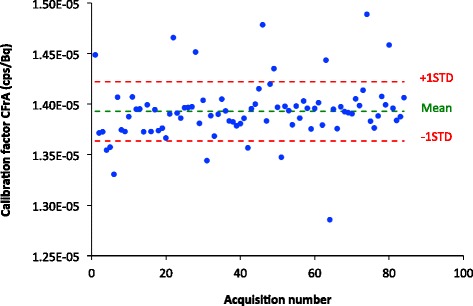
**Calibration factor distribution obtained using the measured calibration activity (CF**_**rA**_ **).**

The mean value of the CF_rA_ was equal to 1.393 × 10^−5^ cps·Bq^−1^, with a standard deviation of 0.032 × 10^−5^ cps·Bq^−1^ (2.3%). The distribution of calibration factor values is shown in Figure 2. Table 2 presents the estimates of the mixed-effects model with camera (*S*_EF_) and radionuclide activity meter (constancy) treated as fixed effects and the background count rate treated as a random effect. These data demonstrate that the effect of camera variations, as assessed by *S*_EF_, on the calibration factor CF_rA_ was very low. Recall that the sensitivity factor is based on the extrinsic flood count rate measured with a ^57^Co sheet source. This factor was included to account for variations in the camera sensitivity that are independent of radionuclide and collimator. The effect associated with ${S}_{\mathrm{EF}}\left(\mathrm{the}\kern0.5em \mathrm{slope}\kern0.5em {\beta}_{S_{\mathrm{EF}}}\right)$ was not statistically significant (i.e., the *p* value for the hypothesis that the slope was different from zero was >0.05). As an example of the magnitude of the effect of changes in the sensitivity factor on the calibration factor, suppose that the sensitivity factor *S*_EF_ (the average sensitivity factor was equal to 7.68 × 10^−5^ cps·Bq^−1^) increased by one standard deviation (0.07 × 10^−5^ cps·Bq^−1^), CF_rA_ would have increased by ${\beta}_{S_{\mathrm{EF}}}$ times the standard deviation of CF_rA_, that is by 0.001 × 10^−5^ cps·Bq^−1^ (0.1%). On the other hand, variations associated with variations in the radionuclide activity meter, as modeled by the constancy, had a larger effect on CF_rA_. The effect associated with the constancy (the slope *β*_Constancy_) was statistically significant (*p* < 0.05) and 4.5 times higher than the camera effect. An error of 1.5 MBq, equal to one standard deviation of the radionuclide activity meter constancy, in the calibration source activity measurement would have decreased CF_rA_ by 2.5%. Regarding the variance, the inter-calibration source component, ${\sigma}_{\mathrm{C}{\mathrm{F}}_{\mathrm{rA}}}^2$, was negligible compared to the intra-calibration source component, ${\sigma}_{\varepsilon}^2$. The inter-calibration source component is the component of variance that is between sources, and the intra-calibration source component is the variation for the repeated measurements at different days for a single source. The background count rate component ${\sigma}_{\gamma_{\mathrm{BkR}}}^2$ was 30 times lower than ${\sigma}_{\varepsilon}^2$. Poisson noise in the calibration source image can account for some of the intra-calibration source component. That is, each time the same calibration source is imaged, a different number of counts would be obtained as the total count is a sample from a Poisson distribution. To investigate this, we estimated the (standardized) variance of the calibration factor CF_rA_ due to the Poisson noise using Equation 3, which was derived using propagation of errors methodology:

**Table 2:** **Results of mixed-effects REML regression for the calibration factor CF**_**rA**_

	**Estimate**	**95% Confidence interval**
Fixed effects		
${\mu}_{\mathrm{C}{\mathrm{F}}_{\mathrm{rA}}}$	0.0714	−0.1213 to 0.2641
${\beta}_{{\mathrm{S}}_{\mathrm{EF}}}$	0.0482	−0.1392 to 0.2355
*β*_Constancy_	−0.2200	−0.4061 to −0.0339
Random effects (weight of the parameter on the total variance)		
${\sigma}_{\mathrm{C}{\mathrm{F}}_{\mathrm{rA}}}^2$	9.14 × 10^−20^ (0.0%)	
${\sigma}_{\gamma_{\mathrm{BkR}}}^2$	0.026 (3.3%)	
${\sigma}_{\varepsilon}^2$	0.760 (96.7%)	

Note that the values above were obtained using standardized variables, i.e., where the mean of each variable in the model was subtracted and the result was divided by the standard deviation of that variable. Thus, the values above are scaleless.

3 $$ \mathrm{Variance}\left(\mathrm{C}\mathrm{F}\right)\kern0.5em =\kern0.5em \frac{\mathrm{Calibration}\kern0.5em \mathrm{source}\kern0.5em \mathrm{count}/\mathrm{T}\mathrm{i}\mathrm{m}{\mathrm{e}}^2\kern0.5em +\kern0.5em \mathrm{Background}\kern0.5em \mathrm{count}/\mathrm{T}\mathrm{i}\mathrm{m}{\mathrm{e}}^2}{\mathrm{Calibration}\kern0.5em \mathrm{source}\kern0.5em {\mathrm{activity}}^2\kern0.5em \times \kern0.5em \mathrm{S}\mathrm{T}{{\mathrm{D}}_{\mathrm{CF}}}^2} $$

Using this, the Poisson noise-estimated variance was equal to 0.025, which represented only 3.3% of the intra-calibration source variance, ${\sigma}_{\varepsilon}^2$. Thus, there was considerable variation in the calibration factor over time for a given patient even though the same calibration source was used.

### Calibration factor obtained by averaging decay-corrected source activities: CF_dA_

**Figure 3:**
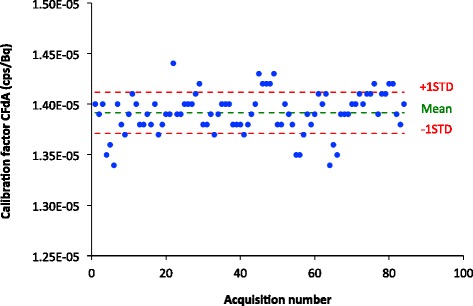
**Calibration factor distribution obtained using the averaged calibration source activity (CF**_**dA**_ **).**

As noted in the previous section, even though the same calibration source was used, there was a significant amount of intra-calibration source variation. One possible source of this variation is errors in the measurement of the activity of the calibration source using the radionuclide activity meter. For example, operators might not have consistently placed the source in the same position in source holder, might not have waited for readings to stabilize, or have made errors in recording the readings. To reduce this source of variation, we averaged the calibration source activities measured for the same source over all the measurement times after decay, correcting the calibration source measurements back to the time of imaging. This average calibration source activity was then used in the calculation of the calibration factor CF_dA_. The distribution of the calibration factor CF_dA_ is shown in Figure 3. The mean of CF_dA_ was equal to 1.391 × 10^−5^ cps·Bq^−1^ with a standard deviation of 0.025 × 10^−5^ cps·Bq^−1^ (1.8%). Thus, averaging the decay-corrected calibration source activity measurements resulted in a 23% reduction in the variability of the calibration factor. The estimated model parameters for the mixed-effects model (model 1) are shown in Table 3, where *S*_EF_ and the constancy were treated as fixed effects and the background count rate was treated as a random effect. Once again, the slope corresponding to the sensitivity factor was not statistically significant (*p* > 0.05) and had a small effect on the calibration factor. For example, if *S*_EF_ increased by one standard deviation, the calibration factor CF_dA_ would have decreased by 0.0004 × 10^−5^ cps·Bq^−1^ (0.03%), meaning, there was a negligible effect of camera sensitivity variations (as measured by *S*_EF_) on the CF_dA_ measurement. The averaging of the decay-corrected calibration source activity measurements reduced the importance of the radionuclide activity meter constancy, but it was still relatively large compared to the influence of camera sensitivity. The calibration factor CF_dA_ would have decreased by 1.7% if the measured activity of the calibration source activity had an error equal to one standard deviation (1.5 MBq). The effect corresponding to the constancy was not statistically significant (*p* > 0.05). The inter-calibration source variance, ${\sigma}_{\mathrm{C}{\mathrm{F}}_{\mathrm{dA}}}^2$ was the main component of the total variance (77%). The intra-calibration source variability, ${\sigma}_{\varepsilon}^2$, represented 14% of the total variance, with 54% of this due to Poisson noise. The variance due to the background was the smallest component (9%).

**Table 3:** **Results of mixed-effects REML regression for the calibration factor CF**_**dA**_

	**Estimate**	**95% Confidence interval**
Fixed effects		
${\mu}_{\mathrm{C}{\mathrm{F}}_{\mathrm{dA}}}$	0.1522	−0.0690 to 0.3734
${\beta}_{{\mathrm{S}}_{\mathrm{EF}}}$	−0.0157	−0.1444 to 0.1759
*β*_Constancy_	−0.1317	−0.2935 to 0.0300
Random effects (weight of the parameter on the total variance)		
${\sigma}_{\mathrm{C}{\mathrm{F}}_{\mathrm{dA}}}^2$	0.4443 (77.4%)	
${\sigma}_{\gamma_{\mathrm{BkR}}}^2$	0.0510 (8.9%)	
${\sigma}_{\varepsilon}^2$	0.0789 (13.7%)	

Note that the values above were obtained from standardized variables, i.e., where the mean of each variable in the model was subtracted and the result was divided by the standard deviation of that variable. Thus, the values above are scaleless.

### Background and calibration source count rates

The statistical analysis using model 2 showed a low coefficient of variation of 1.3% (19.27 ± 0.25 cps) in the background count rate over all acquisitions after excluding the outliers. The intra-measurement variance estimate, ${\sigma}_{\varepsilon}^2$, and the inter-measurement variance estimate, ${\sigma}_{\mathrm{BkR}}^2$, had the same proportion. The Poisson noise variance, estimated using (Count/Time^2^)/STDBkg^2^, represented 74% of the inter-measurement variance.

We analyzed the factors affecting variation in the calibration source count rate using model 3. The mean calibration source count rate was equal to 150.5 cps with a standard deviation of 13.2 cps. The coefficient of variation calculated from these values was thus 8.9%. This was close to the coefficient of variation of the measured calibration source activities over all the vials (excluding the outliers), which was equal to 9.4%. The inter-calibration source variance estimate, ${\sigma}_{\mathrm{SR}}^2$, represented 73.4% of the total variance estimate. The effect of the background count rate variance ${\sigma}_{\gamma_{\mathrm{BkR}}}^2$ on the calibration source count rate was close to zero and very small compared to the inter-calibration source and intra-calibration source variance estimates.

Finally, for both the low count rate of the background and higher count rate of the calibration source, the camera sensitivity (*S*_EF_), treated as a fixed effect, had a non-statistically significant association with a *p* value above 0.05 and a small effect on the calibration factor.

## Discussion

The development of SPECT reconstruction methods including advanced corrections for physical interactions in the patient and detectors has provided the ability to obtain images with absolute activity quantification using SPECT/CT imaging. However, the conversion of voxel values to activity units, which is performed with the use of a calibration factor, is not well standardized and methods to reliably estimate this factor are needed. In this work, we focused on a calibration factor obtained from a planar acquisition of a small calibration source imaged in air due to its relative simplicity. We evaluated the repeatability of this method and studied the factors that affect the variability of the resulting calibration factor. The calibration factor estimate was obtained based on acquisition of an image of a vial containing a measured quantity of the radionuclide of interest before each patient image acquisition. The process of filling a new vial for each patient and the use of a radionuclide activity meter to measure the calibration source activity for each acquisition were potential sources of variability. The constancy test performed as part of daily radionuclide activity meter quality control was used as an indicator of radionuclide activity meter instrumentation variability. The background count rate, another potential source of variability, was measured by a planar acquisition without any calibration source in the field of view and was subtracted from the calibration source count rate. Finally, the calibration factor repeatability could be affected by variations in camera sensitivity or table speed over time. A sensitivity factor calculated from a daily ^57^Co extrinsic flood acquisition, also acquired as part of routine quality control, was used to evaluate the impact of the camera sensitivity variations that are independent of radionuclide and collimator on the calibration factor measurement. In order to estimate the components of the total variance of the calibration factor and to determine the parameters that affect the variability of the calibration factor, a mixed-effects model was used to analyze the data.

The camera response and the radionuclide activity meter variations via, respectively, the sensitivity factor and the constancy were considered fixed effects in the model. According to the results, camera sensitivity variations were small and had little effect on the variance of the calibration factor. The extrinsic flood source count rate was corrected for dead time using a paralyzable model, while current SPECT cameras are neither purely paralyzable nor non-paralyzable [16]. It is possible that errors due to this correction masked the usefulness of the sensitivity factor to explain variations in the calibration factor. Furthermore, the extrinsic flood was acquired with a ^57^Co source and LEHR collimator, while the calibration factor was measured with an ^131^I source and HEGP collimator. Thus, the camera sensitivity factor would not explain variations in the calibration factor that depended on radionuclide or collimator. Nevertheless, these results do not support the use of a ^57^Co source as a predictor of small variations in the calibration factor. However, since the data here did not have instances where there were large variations due to factors such as photomultiplier tube failure, they do not provide information about the utility in such cases. Thus, daily sheet source count rate may still be useful for quantitative quality control.

The statistical analysis showed that radionuclide activity meter variation, as measured by the constancy, had a statistically significant but relatively small impact on the calculated calibration factor. Based on the results, an increase of 1.5 MBq (equal to one standard deviation of the radionuclide activity meter constancy measurement) in the calibration source activity measured with the radionuclide activity meter would result in a decrease of 2.5% in CF_rA_ and 1.7% in CF_dA_.

As mentioned, we used two methods to calculate the calibration factor. The factor CF_rA_ was calculated using the calibration source activity measured with the radionuclide activity meter each day of the calibration source acquisition. The standard deviation over the 84 measurements was equal to 2.3%, and the main component of the variance was the intra-calibration source variance. The inter-calibration source variance component was close to zero. Poisson noise introduced by the count acquisition process should ideally comprise the majority of intra-calibration source variance, but represented only 3.3% of the variance. This indicated that variability due to measurement of the same calibration source was a significant source of error. This suggests that careful and repeated measurement of calibration source activity is an important way to reduce calibration factor variability.

Averaging the calibration source activity over measurements made on different days resulted in reduced importance of the intra-source variance. The most important variance components for the calibration factor CF_dA_, which used the average of the measured calibration source activity over the three time points, were the inter-calibration source (77%), intra-calibration source (14%), and background count rate components (9%). Approximately 54% of this intra-calibration source variation was explained by variance due to Poisson noise, which could be reduced by longer calibration source image acquisition times or higher calibration source activities. The use of the average of the calibration source activity decreased the standard deviation of the calibration factor to 1.8%, a 23% reduction, and decreased the effect of radionuclide activity meter variability on the calibration factor estimate. This indicates that the radionuclide activity meter should not be considered as a simple fixed effect but as a combination of fixed and random effects. A part of the random component may be due to the variable positioning of the calibration source inside the radionuclide activity meter leading to variability of the calibration source activity measurement [15]. The remaining variance in the intra-calibration source component may be due to the variability of the bed speed during the whole-body acquisition, the calibration source position in the camera field of view, the calibration source position on the bed, or variations in the distance between the calibration source and the detectors. Imprecision or inaccuracy in the manually recorded time of the calibration source activity measurement or unsynchronized clocks between the radionuclide activity meter and the camera may also explain some of this intra-calibration source variance component.

Another potential source of intra-calibration source variability is difference in dead-time losses at the different time points due to the differences in source strength. For example, the count rate 6 days after source preparation (day 6) would be 60% of the count rate on the day of preparation (day 0). Thus, the count rates on day 0 would be affected more by dead-time effects, and the decay- and background-corrected count rates from day 8 would be larger than those from day 0. However, the average count rates for the calibration sources in these experiments were on the order of 150 cps, and the count rate losses are thus expected to be very small. To verify this, we computed the difference divided by the mean (i.e., the relative difference) between the background- and decay-corrected count rates for each pair of time points for each patient. To be specific, we computed these relative differences between the count rates for time points 1 and 2, 2 and 3, and 1 and 3 after background correction and dead-time correction to the time of acquisition for point 1. If dead-time effects were important, then we would expect systematic differences in the relative differences in the count rates for the various pairs of time points. For each of these pairs, we computed the average and standard deviations of the relative differences and tested the null hypothesis that these relative differences were non-zero. The average relative differences were all small (<0.11%) and the differences were not statistically significant (*p* > 0.5 for all three cases). This confirms that count rate effects were too small to explain the observed intra-calibration source variability.

The largest source of variability in the calibration factor CF_dA_ was the inter-calibration source variance component, representing 77% of the total variance. Thus, understanding and reducing this source of variance could substantially reduce overall calibration factor variability. This represents variance in the calibration factor that varied with different sources. One likely source of this variation is preparation of the calibration source. For example, using different volumes in the source could affect both the activity measured with the activity meter and the gamma camera count rate (due to self scatter and attenuation in the source). Longer-term variations of the sensitivity that were dependent on the collimator or radionuclide, and thus not captured in the camera sensitivity factor, would also result in inter-calibration source variability. One way to distinguish between these potential sources would be to use a sealed source with a long half-life having similar energy photons as ^131^I and imaged with the same collimator. A ^133^Ba source might be useful for this application.

Based on these results and observations, recommendations and suggestions can be made when a planar acquisition of an in-air small calibration source is the basis for determining the calibration factor.

1. Careful measurement of the activity in the calibration source is essential. Considering the statistically significant effect of the radionuclide activity meter on the calibration factor measurement, consideration should be given to using the average of repeated measurements or a more accurate and precise activity measurement device.
2. Care in controlling the background radiation environment is essential. Because the variance estimate due to the background count rate was non-negligible for the calibration factor CF_dA_, particular attention should be taken to avoid sources of activity near the camera during the calibration source and patient acquisitions in order to keep the same level of background counts. Another way to reduce the impact of background radiation on calibration factor variability would be to use a region of interest over the calibration source rather than simply the counts in the entire image. However, care in defining the region would be needed to avoid adding variability to the measurement.
3. The calibration factor should be determined carefully less frequently with more frequent checks on its stability. The camera variability as measured by the sensitivity factor, which was very low over time, had a very small effect on the final calibration factor measurement. There was some significant residual variance in the calibration factor measurement that was not explained. Possible sources include variation in the placement of the calibration source. As a result, careful calibration source measurement, including use of carefully controlled calibration source volumes and position in the imaging field of view, would seem desirable. In addition, calibration using a SPECT/CT acquisition may be desirable, though was not investigated here. Both of these would require more care in the calibration source acquisition. However, given the level of observed stability in the camera sensitivity and calibration factor, it seems that it is not necessary to repeat this calibration for each patient. Instead, quantitative monitoring of the camera count rate during routine QC or less careful calibration source acquisition using a planar acquisition could be performed for each patient or each morning to check for large changes in the camera sensitivity and as an indicator that recalibration is needed. For SPECT calibration, acquisition of a static planar acquisition instead of a whole-body scan would eliminate another potential source of variation, bed speed variability. Further investigation is needed to more completely assess the effect of these factors on the calibrator factor estimate.

## Conclusions

This study investigated the factors affecting the variability of the calibration factor estimated using a method based on planar imaging of a small in-air calibration source, a method applied clinically for planar dosimetry in a number of therapy applications. Based on these data, using a calibration source that was filled for each patient and imaged before each acquisition resulted in a variability of 2.3% over the 4-year period investigated. When the decay-corrected mean activity averaged over the three time points for each patient was used, the calibration factor variability decreased to 1.8%. Using statistical analysis based on a mixed-effects model, we investigated the factors that contributed to this variability. The largest source of variability was the measurement of the calibration source activity in the radionuclide activity meter. The second most significant source was the background activity. The camera sensitivity measured with a ^57^Co sheet source was very stable with time and did not contribute significantly to the overall variability. However, this sensitivity would not account for variations that are radionuclide dependent. These data suggest that careful preparation of the calibration source and measurement of its activity and controlling the background environment would improve calibration factor repeatability. Considering all these factors, less frequent careful calibration combined with more frequent but less exacting checks on camera sensitivity may be the most clinically practical method to obtain reliable activity estimates from nuclear medicine images.
